# Old growth forests and large old trees as critical organisms connecting ecosystems and human health. A review

**DOI:** 10.1007/s10311-021-01372-y

**Published:** 2022-01-05

**Authors:** Melinda Gilhen-Baker, Valentina Roviello, Diana Beresford-Kroeger, Giovanni N. Roviello

**Affiliations:** 1 Faculty of Physical Medicine and Rehabilitation, Georgian State Teaching University of Physical Education and Sport, 49, Chavchavadze avenue, 0162 Tbilisi, Georgia; 2 Department of Chemical, Materials and Industrial Production Engineering (DICMaPI), University of Naples Federico II, Piazzale V. Tecchio 80, 80125 Naples, Italy; 3 Department of Biology, Carleton University, 1125 Colonel By Drive, Ottawa, ON Canada; 4 Istituto Di Biostrutture e Bioimmagini, IBB - CNR Mezzocannone Site and Headquarters, 80134 Naples, Italy

**Keywords:** Old-growth forests, Large old trees, Carbon sequestering, *Fomitopsis officinalis*

## Abstract

Old forests containing ancient trees are essential ecosystems for life on earth. Mechanisms that happen both deep in the root systems and in the highest canopies ensure the viability of our planet. Old forests fix large quantities of atmospheric CO_2_, produce oxygen, create micro-climates and irreplaceable habitats, in sharp contrast to young forests and monoculture forests. The current intense logging activities induce rapid, adverse effects on our ecosystems and climate. Here we review large old trees with a focus on ecosystem preservation, climate issues, and therapeutic potential. We found that old forests continue to sequester carbon and fix nitrogen. Old trees control below-ground conditions that are essential for tree regeneration. Old forests create micro-climates that slow global warming and are irreplaceable habitats for many endangered species. Old trees produce phytochemicals with many biomedical properties. Old trees also host particular fungi with untapped medicinal potential, including the Agarikon, *Fomitopsis officinalis*, which is currently being tested against the coronavirus disease 2019 (COVID-19). Large old trees are an important part of our combined cultural heritage, providing people with aesthetic, symbolic, religious, and historical cues. Bringing their numerous environmental, oceanic, ecological, therapeutic, and socio-cultural benefits to the fore, and learning to appreciate old trees in a holistic manner could contribute to halting the worldwide decline of old-growth forests.

## Introduction

**Fig. 1:**
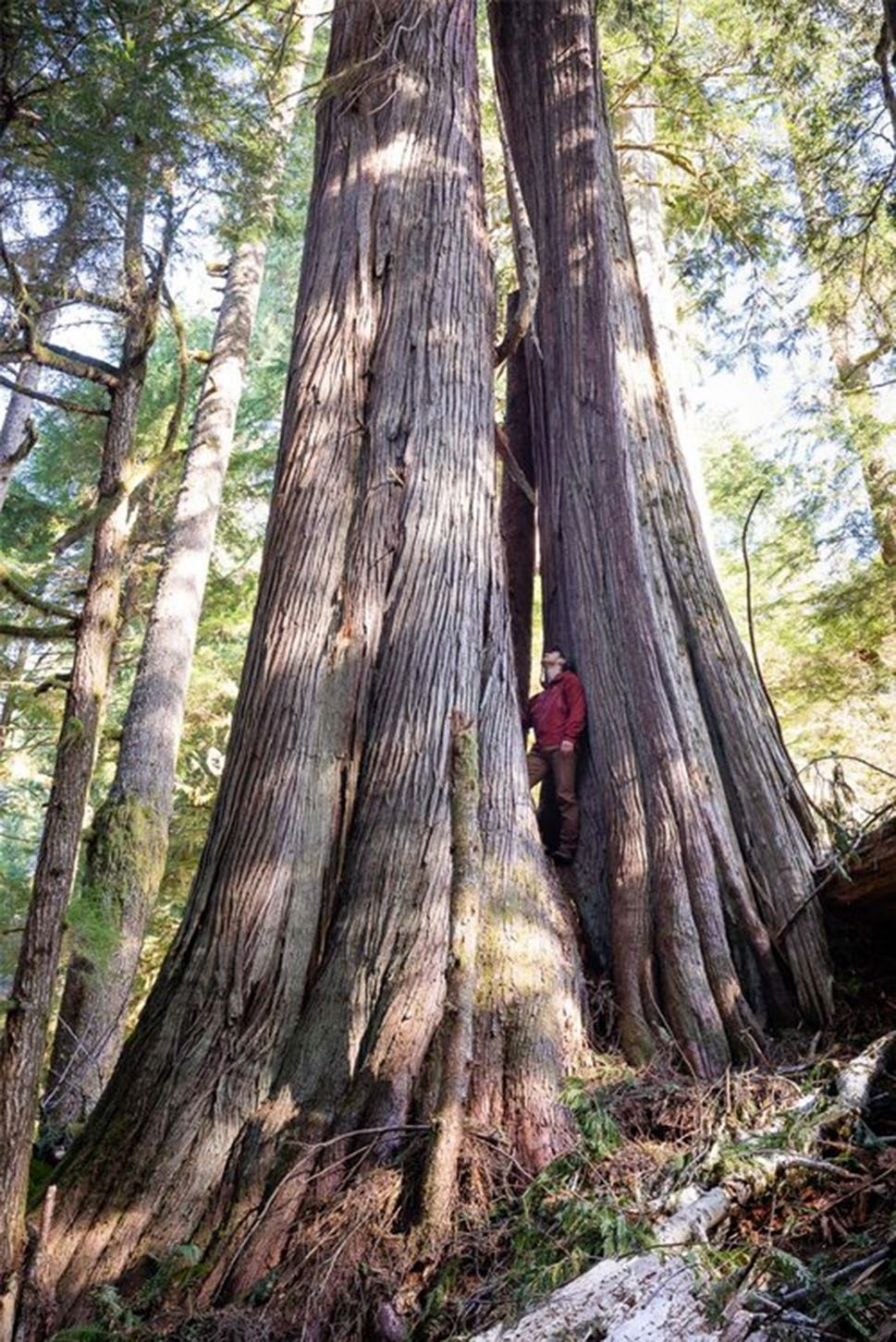
The great Arborvitae, the tree of life. Note its size and girth when compared to the photographer, as well as the many creases and holes in the buttress system. These trees are ideal homes for many species and are only present in older trees. Apart from being an ideal habitat for many species, large old trees such as this one continue to sequester carbon while producing large amounts of beneficial aerosols. This particular specimen of Western redwood, *Thuya plicata,* was photographed by TJ Watt in the old-growth forests of Vancouver Island, British Columbia, Canada

Ancient trees (Fig. 1) have captured the imagination of humankind since time immemorial, and have become symbols of health and medicine, family, and even life itself. As literal and figurative pillars to many ecosystems on our planet, trees continue to provide oxygen, wood, food, and medicines while purifying our air by sequestration and filtration (Beresford-Kroeger 2018). Forests also contribute to the ecology of our oceans by providing the necessary iron for the replication of *Cyanophyta* (Matsunaga et al. 1982; Deein et al. 2002; Krachler et al. 2019). It is not surprising then that in recent years there has been an increased interest in the conservation of the last stands of old-growth forests around the globe with more and more studies into their particular traits (Spies 2004). One barrier faced to the protection of old growth forests is the difficulty they present in defining them (Spies 2004; Frelich and Reich 2003). The variety found between species of older trees, their height, diameter, and lifespan, for example, is quite stark which makes the creation of a definition which would be transferable between species and ecosystems very difficult (Lindenmayer and Laurance 2016). This lack of clear definition also makes the creation of guidelines for their protection tricky and makes old-growth forests and their large old trees susceptible to unchecked logging. Due to the high quality of their wood, it isn’t surprising that many of the world’s largest, straightest and healthiest trees have already been culled, with some, such as the bur oak (*Quercus macrocarpa*) of North America being reduced to an inferior retrograde status, both stunted and twisted (Beresford-Kroeger 2003).

Forests around the world are responsible for a large part of the oxygen so essential to our survival (Beresford-Kroeger 2018; Huang et al. 2018) but old-growth forests have often been left out of the equally important carbon sequestration equation. Until recently, it was believed that they no longer pulled carbon out of the air but current findings have shown that to be untrue (Schulze et al. 2009). On top of continuing to pull carbon from our atmosphere and accounting for at least 10% of the worlds carbon sequestration capabilities (Luyssarert et al. 2008), disturbing these great stands of trees releases the considerable carbon stores that they would otherwise hold fast both in their bodies and in the soil beneath them. In losing old-growth forests, we would also lose their nitrogen-fixing capabilities. Moreover, large old trees have the particular ability to nurture the surrounding saplings; thus helping to ensure the health of the entire forest. This hospitality is extended to many species of endangered animals for which old-growth forests create important microclimates and ideal living conditions. Of course, old trees are also of great interest to human health. Apart from providing medicinal compounds themselves (Beresford-Kroeger 2018), they are host to mushroom populations which hold untapped potential for future pharmaceuticals (Stamets 2005) such as possible COVID-19 treatments and new antibiotics. As already alluded to, the magnificence of ancient trees has been a source of inspiration to cultures worldwide and continues to be a deeply engrained part of our very identity as human beings.

## Carbon sequestration

### Continued carbon sequestration through growth and reduced respiration

With the current global average of atmospheric carbon dioxide measuring nearly 420 parts per million, representing a 12% increase since the year 2000, ensuring we lower emissions and increase sequestration is vital (Lindsey 2020; Ali and Khan 2017). With this in mind, old-growth forests continue to accumulate carbon at a much greater rate than was previously thought, making them important carbon sinks which researchers say must be factored into global climate models (Schulze et al. 2009). Until recently, it was assumed that older forests no longer absorbed carbon due to their slower growth rate (Schulze et al. 2009; Luyssarert et al. 2008). With new growth only occurring in the small spaces left by the death and decomposition of older trees, which in turn release their accumulated carbon, old-growth forests were considered to be carbon neutral and thought of as such in climate models (Schulze et al. 2009). In the past decade or so, murmurs of disagreement over this concept have surfaced and individual projects have found that even very old forests are capable of storing carbon due to tree growth, the addition of new trees, and a decreased rate of respiration in old trees (Schulze et al. 2009; Dimri et al. 2017). Indeed, their carbon sequestering capabilities may even be greater than younger plantation-type forests (Yang et al. 2021). Starting in the mid-1990s, global forest carbon fluxes have been measured using much more sophisticated means with data being shared between members of Fluxnet (a global network of ecosystem and atmospheric trace gas flux measurement sites) (Luyssarert et al. 2008). Using this data, a meta-analysis was produced which looked at 519 areas of temperate and boreal forests aged between 15 and 800 years old which concluded that carbon is still being absorbed by the world’s old-growth forests and these particular eco-systems account for about 10% of the world’s carbon sequestration capability (Luyssarert et al. 2008).

### Unaccounted carbon storage within the soil of old-growth forests

The fact that ancient trees offer much more than simply planks of wood and that their value is far greater while alive is becoming harder and harder to ignore. The old boreal forests are indeed vital carbon sinks that maintain billions if not many more tons of carbon dioxide in the phenolic acid of their soil. If they were to be disturbed, be made to feel the brunt of the axe, this would release untold amounts of carbon into the increasingly carbon saturated atmosphere (Beresford-Kroeger 2013). Adding another layer to this story is the fact that under the canopy of these great boreal forests, from Canada to China and Russia, there is a “second forest” consisting mainly of nitrogen-fixing lichens. These not only help to retain large amounts of carbon in the soil but also feed the migrating caribou (*Rangifer tarandus*, conversation with Diana Beresford-Kroeger, November 17th, 2021). WWF Canada has also just released a detailed report, submitted to the Global Biogeochemical Cycles, which demonstrates that the soil organic carbon of the great boreal forests was widely underestimated. It also supports the carbon storage capacity of large old trees from the old-growth forests of the Pacific Maritimes eco-zones (Sothe et al. 2021). This leads to the fact that trees do not only store carbon in their woody bodies, their extensive root systems, and their accompanying mycorrhizae, but they also create the right conditions in the soil for it to also become an important carbon trap (conversation with Diana-Beresford Kroeger, November 19th, 2021). Large amounts of carbon can also be trapped in the soil directly under the trunks of large old trees and within their buttresses, a fact ignored by many models approximating soil organic carbon (Dean et al. 2020). Indeed, it has been found that old-growth forests are more effective at holding carbon in the soil due to richer organic matter and their particular chemical composition (Xiong et al. 2021). Both carbon and nitrogen cycling have thus been found to be affected by the abundance and diversity of the microbes present in the soil (Louis et al. 2016). In one particular study done in subtropical China, it was found that old-growth forests have a lower soil organic carbon turnover rate than do younger forests due at least in part to decreased pH and higher nitrogen concentration which together slow microbial growth and reduce soil respiration (Xiong et al. 2021). The capacity for old-growth forests to trap large amounts of carbon even within their soil and the fact that this carbon would be released if the forest is disturbed must also be factored into any decision on whether or not to harvest these trees.

## Nitrogen fixation factories

Where does the increase in nitrogen we have mentioned come from in areas where there seems to be little of this precious nutrient available such as certain old-growth forests of western Canada? This too can be answered by particularities of old-growth forest ecology. This time one must look up to the canopies to find particular algae, bryophytes, ferns, and lichens. One specific study done in the Pacific North Western temperate rain forest looking at old growth Sitka spruce (*Picea sitchensis)* trees, in particular, demonstrates that most of its stand-level biological nitrogen fixation occurs within epiphytic bryophyte systems and lichen populations which house N-fixing cyanobacteria (Lindo and Whiteley 2011; Brodo et al. 2001). Another study, this time comparing second growth and old growth grand fir (*Abies grandis)* forests in northwestern Montana, showed that N-fixing foliose lichens were much more abundant in the older forests than in the younger, with certain species being completely absent from the newer stands of trees (Lesica et al. 1991). With many beneficial mosses, lichens, and liverworts needing particular conditions to grow, conditions which are often specific to old-growth forests (Lesica et al. 1991; Malicek et al. 2019), it is almost certain that with the loss of these old trees, we will also be losing many other organisms along with their many benefits including their nitrogen fixation capabilities.

## Microclimates and safe havens for species at risk from global warming

As climate change (Han et al. 2021; Zheng et al. 2020) continues to affect ecosystems worldwide, finding ways to slow the rise in temperatures and help local fauna cope with these changes is becoming critical as biodiversity is increasingly threatened. In 2018 alone, the world was faced with 315 cases of climate-related natural disasters 26 of which were incidences of extreme temperatures (Fawzy et al. 2020). In this context, old-growth forests, and their ability to create microclimates, are of great interest. Indeed, when compared to second-growth plantation forests, old-growth forests with their denser and more complex biomass and higher canopies have been found to provide cooler microclimates (Frey et al. 2016). In one such study done in Oregon, USA, the difference between temperatures taken from comparable first growth and second growth sites was as much as 2.5 °C (Frey et al. 2016). Similar results were found in studies comparing temperatures in old-growth and second-growth forests in the UK as well as the Ukraine (Norris et al. 2011). Old-growth forests then become an important refuge site for a multitude of species which are particularly sensitive to rises in temperature and allow them the time to adapt (Frey et al. 2016; Wolf et al. 2021; Betts et al. 2017). This ability to keep warm temperatures at bay through the creation of microclimates is a critical piece to mitigating the effects of climate change for animals, plants, and humans alike.

## Essential habitats for endangered species.

Large old hollow-bearing trees in particular, with their deep craggy bark, crevices, and boles which come with age, also provide habitat for different plant and animal species which younger trees and artificial structures cannot replace (Lindenmayer et al. 2013; Le Roux et al. 2018). This is true even for single or small groups of large old trees marooned in the landscape (Lindenmayer 2017). The fate of one of the most critically endangered animals in Canada, the spotted owl (*Strix occidentalis*), is intrinsically linked to that of the old-growth forests of British Columbia where it makes its home. Many other vertebrates and vascular plant species are also at risk of extinction or extirpation due largely to logging activities in the woods of British Columbia (Yezerinac and Moola 2006). In other old-growth forests of the Pacific Northwest, this time in the United States, the Olympic salamander (*Rhyacotriton olympicus*), the Del Norte salamander (*Plethodon elongatus*), and the tailed frog (*Ascaphidae*) were all found to rely primarily on these particular eco-systems for their survival (Welsh 1990). With species from both the top and bottom of the food chain at risk in the diminishing old-growth forests, the cascade effect on losing them forever is hard to predict but could certainly be severe. Around the world, many other species have suffered due to the loss of large old trees such as the orangutan (*Ponginae*) in southeast Asia and the lead-beaters possum (*Gymnobelideus leadbeateri*) in south-east Australia (Jones et al. 2017). We have already lost many species of both plants and animals completely due to the destruction of native forests which are very difficult and sometimes impossible to restore (Miyawaki 2004). This is why conserving the remaining old-growth forests is essential to limit the losses of yet more endangered plants and animals which otherwise would have nowhere else to go (Miyawaki 2004).

## Parental figures for the next generation of trees

### Large old trees offer superior genetics to their progeny

Many studies have started looking at the vast and intricate economic and informational network that lives below the forest floor (Simard et al. 2012; Van Der Heijden and Horton 2009). The root system combined with bacteria and mycorrhizae is indeed a busy and complex arena essential to the survival of each part together as a whole. Although each plays a role, large old trees, dubbed “mother trees” by Diana Beresford Kroeger, hold the knowledge and ability to ensure the success of their progeny as well as that of the entire forest (Beresford-Kroeger 2011). They are also endowed with the best genetics and medicinal arsenal for the benefit of all (Beresford-Kroeger 2011). Indeed, old-growth forests have been found to contain the most stress-resistant specimens of trees as well as trees that have best learned to deal with competition (Norris et al. 2011; Ammar et al. 2020). Large old trees also often have interesting genetic variations which give them the ability to survive extremes in climate and competition with other species (Frelich and Reich 2003). With the loss of this important gene pool, new generations will be inferior and less hardy such as the bur oak of North America (*Quercus macrocarpa*) which we have previously mentioned. A similar fate also befell the North-American white pine (*Pinus strobus).* Being particularly susceptible to the loss of genetic diversity due to logging, most white pine which have been planted as second-growth are stunted, only reaching a height of 25 m, half of what their older relatives can grow to be (Frelich and Reich 2003). Retaining the remaining large old trees to ensure the genetic viability of future generations of trees is thus crucial to the survival of our forests.

### Large old trees create the ideal soil conditions for the next generation to grow

Since both carbon and nitrogen-containing nutrients are essential, it is once again relevant to use these as markers to demonstrate how large old trees are contributing to their surroundings. It has been found that larger trees make use of the mycorrhizal network to send carbon to smaller trees which might not have as good access to sunlight (Beresford-Kroeger 2011). By investigating quantities of stable isotopes of both carbon and nitrogen measured in the wood and surrounding soil of large old pines and nearby younger trees, a study done on the native Scots pine (*Pinus sylvestris*) forests of Great Britain also demonstrated how large older trees create ideal nurseries for younger growth to flourish (Weber et al. 2008). It is suggested that carbon and nitrogen transference occurs through foliar carbon and nitrogen source-sink gradients or perhaps these nutrients move as free amino acids through the mycorrhizal network or maybe by a combination of these (Teste et al. 2009). It is clear however that seedlings have a much lower mortality rate when planted near older donor trees (Teste et al. 2009). They also have a much faster growing rate when they are planted near older mother trees (Frelich and Reich 2003). Ensuring the survival of the older and larger trees with the best genetics is then essential for the continuing success of the wider forest and will certainly be critical for forest restoration projects.

## Medicinal powerhouses

### Forest bathing and its proven therapeutic benefits

Plants certainly provide numerous benefits for the health of the environment but this is also true when looking at their medicinal potential for human health (Crini et al. 2020). In a previous review, we touched on “Shirin Yoku” or forest bathing; an ancient practice involving spending time around trees inhaling the particular aerosols and organic volatile compounds that have many proven therapeutic properties (Li 2010; Roviello et al. 2021). In Japan, as in many places where this practice has survived through the years, this is traditionally done in old-growth forests as much as possible to have the best outcome (Beresford-Kroeger 2018; Li 2010). Of the many possible therapeutic uses for forest bathing, one that stands out in current global events is its use against COVID-19 (Dai et al. 2021; Ufnalska and Lichtfouse 2021; He et al. 2021; Khan et al. 2021). This is thanks to the benefits that biogenic volatile organic compounds emitted by trees can have on the body’s immune system and in terms of their antiviral activity (Roviello et al. 2021; Roviello and Roviello 2021). This disease which has resulted in the current pandemic has been the cause of 5,197,692 deaths worldwide as of the 25th of November 2021 (https://www.worldometers.info/coronavirus/). In order to slow the progression of the novel coronavirus SARS-CoV-2 and to prevent new such pathogens from making their appearance in the future, the part that old-growth forests play in preserving our health must be factored into forest management plans (Roviello et al. 2021).

### Large old trees as hosts to endogenous *fungi* with high therapeutic potential

#### *Fomitopsis officinalis* as a possible treatment for COVID-19

**Fig. 2:**
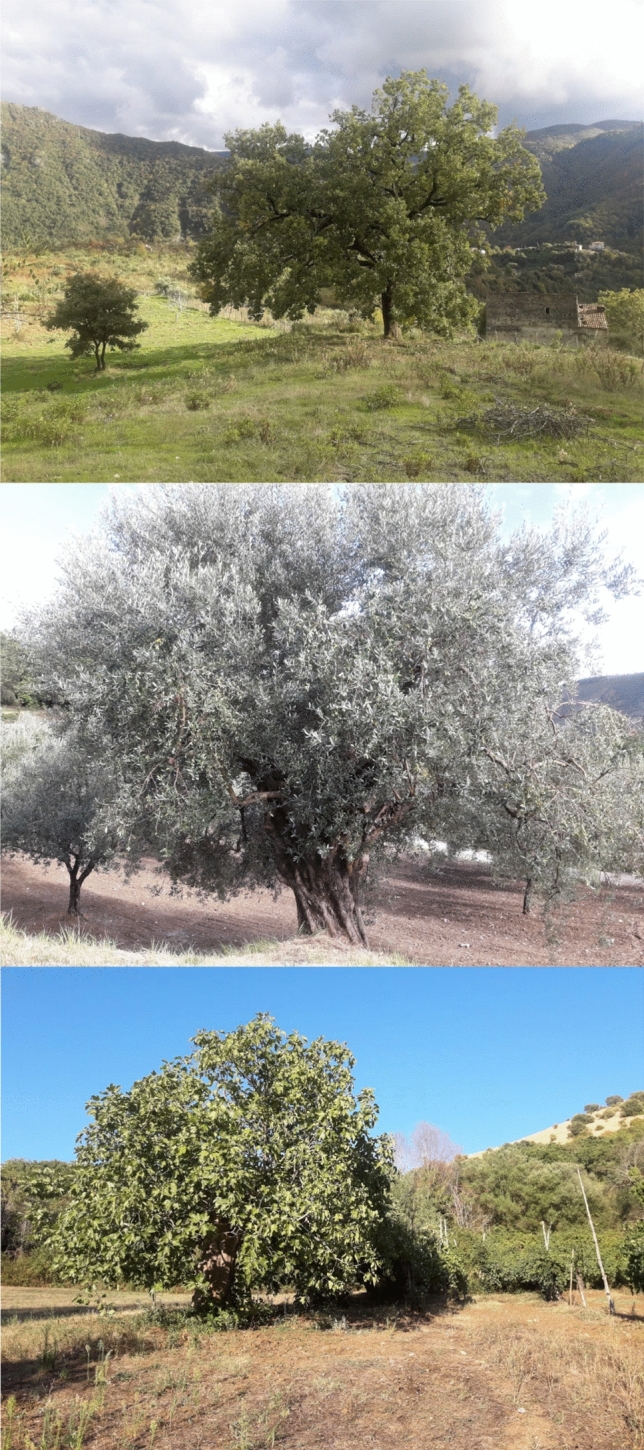
Wild and cultivated old trees from Europe: a large old European oak (*Quercus robur*, up); a large old olive tree (*Olea europaea*, middle), and a large old fig tree (*Ficus carica*, down). These photographs, when compared to Fig. 1, also demonstrate the difficulty in defining large old trees depending on interspecies characteristics. Although set apart, old singular trees contain superior genetics and have the potential to help create the forests of the future. Photos taken in Papasidero, Calabria (European oak) and Castel di Sasso, Campania (olive and fig trees), South Italy (photos courtesy of the Roviello family)

On top of the medicines provided directly from old trees (Fig. 2), there is another quiet inhabitant of ancient forests that shows great potential for human health. The Agarikon mushroom, *Fomitopsis officinalis,* has been used in countries around the world for thousands of years; its wide range of benefits are still being researched and are yet to be fully developed (Stamets 2005). The studies that have been done so far have reported Agarikon as having antibacterial, anticancer, antiviral, anti-inflammatory, and antituberculosis activities (Elkhateeb et al. 2019; Girometta 2019). Unfortunately, this particular mushroom grows very slowly and is increasingly difficult to find which has encouraged its cultivation in the boreal forests (Elkhateeb 2020). In recent pre-clinical studies, Agarikon has been shown to inhibit influenza A (H1N1), cowpox, and herpes among other viruses (Slomski 2021). Together with the fact that they have many antimicrobial properties against bacteria, this has led to the upcoming investigation into the potential this mushroom might have to impede the viral replication of SARS-CoV-2 (Slomski 2021). Here again, large old trees can be a source, even if indirectly, of medicines that can help us in the fight against the current pandemic. As we continue to discover yet more benefits bestowed on the world by trees, it is increasingly evident that old-growth forests and ancient trees are of particular interest for the continued health and wellbeing of our planet and since they are disappearing at an alarming rate, should be protected at all cost.

#### *Fomitopsis officinalis* as a potential source for new antibiotics

The Agarikon mushroom may also have many other tricks up its sleeve due to certain particularities. The American mycologist, Dr. Paul Stamets along with Dr. Pam Kryskow are currently attempting to collect hundreds of strains of Agarikon to conduct a full genome sequencing. This mushroom is of particular interest as it is the longest living species of fungus on earth, at 75 years it outlives many humans, and it has learned to defend itself against countless strains of bacteria (Stamets et al. 2019). The particular phytochemicals which endow Agarikon with such a strong immune system are just one of the components that makes these mushrooms of interest from a medical point of view (Stamets et al. 2019). Much like the isolation of *Penicillium chrysogenum* which led to penicillin, isolating the most active strains of *Fomitopsis officinalis* may produce new lifesaving antibiotics (Stamets 2005). Indeed, when tested against other medicinal mushrooms, ethanolic extracts of *Fomitopsis officinalis* were shown to have the best antimicrobial activity against the two Gram-positive bacteria and two Gram-negative bacteria being observed. In fact, it was the only mushroom tested to show activity against all four strains (Hleba et al. 2016). With new diseases such as COVID-19 making their appearance and with more and more viruses escaping antiviral drugs, the discovery of novel bioactive natural compounds is of particular contemporary interest (Frieri et al. 2017; Slomski 2021).

#### Bioactive compounds isolated from Agarikon (*Fomitopsis officinalis*)

The biomedical properties shown by both crude extracts and the compounds isolated from *Fomitopsis officinalis* fruiting bodies depend on the unique composition of its bioactive compounds, such as coumarins, organic acids, phenolic compounds, polysaccharides, and triterpenoids (Muszynska et al. 2020).

**Fig. 3:**
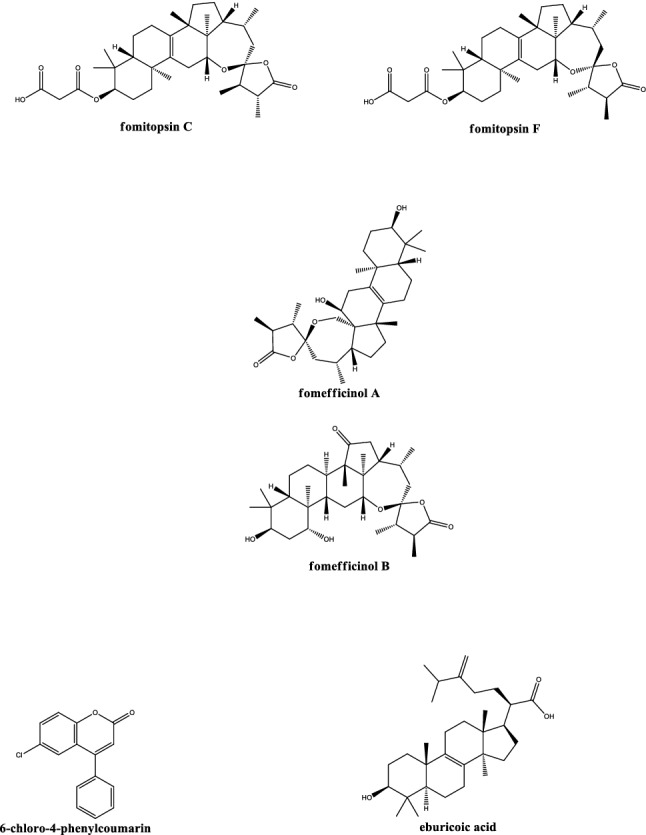
Chemical structures of some phytochemicals isolated from *Fomitopsis officinalis*. Note how fomitopsin C and F, as well fomefficinol A and B were specifically identified from extracts of the *Fomitopsis officinalis*. Other isolated compounds such as eburicoic acid and 6-chloro-4-phenylcoumarin are endowed with anti-inflammatory and antimicrobial properties. Chemical use names (bold) with the corresponding IUPAC names: **fomitopsin C** 3-[(1S,3'R,4'R,5R,7R,10S,13R,15S,17R,18R,21R)-1,3',4',6,6,10,17,21-octamethyl-5'-oxospiro[14-oxapentacyclo[11.7.1.02,11.05,10.018,21]henicos-2(11)-ene-15,2'-oxolane]-7-yl] oxy-3-oxopropanoic acid; **fomitopsin F** 3-[(1S,3'S,4'S,5R,7R, 10S,13R,15R,17R,18R,21R)-1,3',4',6,6,10,17,21-octamethyl-5'-oxospiro[14-oxapentacyclo[11.7.1.02,11.05,10.018,21]henicos-2(11)-ene-15,2'-oxolane]-7-yl]oxy-3-oxopropanoic acid; **fomefficinol A** (1R,2S,3'S,4'S,5S,8R,10R,14S,17R,18R,20S)-2,8-dihydroxy-3',4',5,9,9,14,18-heptamethylspiro[21-oxapentacyclo[12.8.0.01,17.04,13.05,10]docos-4(13)-ene-20,5'-oxolane]-2'-one; **fomefficinol B** (1S,2R,3'S,4'S,5S,7R,9R,10R,11S,13R,15S,17R,18R,21R)-7,9-dihydroxy-1,3',4',6,6,10,17,21-octamethylspiro[14-oxapentacyclo[11.7.1.02,11.05,10.018,21]henicosane-15,5'-oxolane]-2',20-dione; **6-chloro-4-phenylcoumarin** 6-chloro-4-phenyl-2H-chromen-2-one; **eburicoic acid** (R)-2-((3S,5R,10S,13R,14R,17R)-3-hydroxy-4,4,10,13,14-pentamethyl-2,3,4,5,6,7,10,11,12,13,14,15,16,17-tetradecahydro-1H-cyclopenta[a]phenanthren-17-yl)-6-methyl-5-methyleneheptanoic acid

**Fig. 4:**
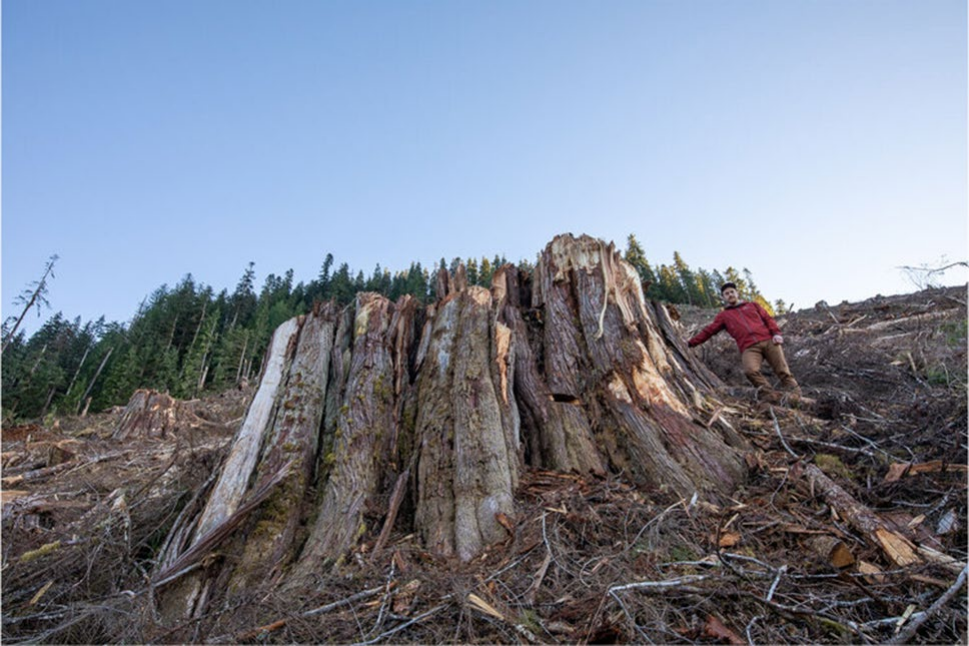
The giant stump of a recently harvested tree of life or Western redwood, *Thuja Plicata,* and the surrounding destruction left by the logging industry. Note again the sheer size of these buttresses and the extent of the root system beneath which are able to hold large amounts of carbon-rich soil. Without this structure, the soil, and its carbon, is at risk of washing away with the increasing floods. This photograph was taken by TJ Watt in the old-growth forests of Vancouver Island, British Columbia, Canada

In Fig. 3 the chemical structures of some specific phytochemicals found in *Fomitopsis officinalis* such as fomefficinol A and B, and fomitopsin C and F (anticancer and antiviral, respectively) are shown together with those of other isolated compounds endowed with anti-inflammatory (eburicoic acid) and antimicrobial (6-chloro-phenylcoumarin, Fig. 3 and Table 1) properties (Muszynska et al. 2020). Almost extinct in Europe and Asia, specimens of Agarikon can still be found in the old-growth forests of both British Columbia and the North-Western United States (Stamets et al. 2019). Protecting its preferred habitat is then essential to its survival and, thus, to the survival of its medicinal gifts. Indeed, our old-growth forests should also be considered as great stores of untapped novel medicines that are of high socio-economic value, yet another valuable argument that they are worth more to us standing than logged (Stamets 2005) (Fig. 4).

**Table 1:** Main compounds from *Fomitopsis officinalis* with the respective therapeutic properties (Muszynska et al. 2020)

Compound name	Therapeutic properties
fomefficinol A	Anticancer
fomefficinol B	Anticancer
fomitopsin C	Antiviral
fomitopsin F	Antiviral
eburicoic acid	Anti-inflammatory
6-chloro-phenylcoumarin	Antimicrobial

## Social and cultural implications for conservation policies

### A living connection to the past

Above and beyond their many ecological benefits, large old trees also provide countless socio-cultural benefits which should not be overlooked when considering conservation policies (Blicharksa and Mikusinski 2014). Trees, and especially the majestic presence of older specimens, can be found as important features in our modern literature, art, and cinematography, often being associated with kings, nobility, mystery, and magic. This more contemporary expression of our fascination with these organisms is but a small glimpse of the long-running and complex relationship people have had with old trees in cultures all over the world (Schweiger and Svenning 2019). With a lifespan crossing many human generations, old trees become a shared living memory, connecting people to their ancestors (Blicharksa and Mikusinski 2013). The second oldest European script, the Ogham, was inspired by trees and the importance they had in the Celtic world (Beresford-Kroeger 2018). Each letter representing a different tree such as the iconic Oak which, as it ages, produces what the druids called *uisce dubh* or “black water” containing gallotannins which are still used today in the treatment of burns (Beresford-Kroeger 2019). Apart from the Celts, ancient indigenous cultures from every corner of the globe have seen trees as connections to the divine with the oldest trees being revered as sacred ancestors (Dafni 2006). Our common history is then intimately connected to large old trees whose value transcends their more tangible benefits.

### Sources of inspiration

The multi-faceted connection to trees has endured throughout the ages. Indeed, modern-day indigenous healers also use a variety of trees in their medicinal tonics whose recipes have been passed down through generations. Ethnobotanist Nancy Turner researched the many medicinal uses of the barks of a total of 23 trees such as the Douglas Fir (*Pseudotsuga menziesii*), the Madrone (*Arbutus menziesii*), and the Western Hemlock (*Tsuga heterophylla*) out of the old-growth forests of Southern Vancouver Island by Saanich and Cowichan Coast Salish people who also rely heavily on the western red-cedar (*Thuja plicata*) for wood and fibers (Turner and Hebda 1990). Their relationship to these great trees is also a spiritual one as through their language they endow trees with anthropomorphic qualities (Turner et al. 1998). Another study was conducted in British Columbia, this time looking at the wider values associated with the old-growth forests by their surrounding inhabitants. The results found that apart from life-support values such as providing clean air, water, and retaining carbon, more than half of all respondents also declared old-growth forests as having aesthetic value and more than 30% felt they also held spiritual value (Connell et al. 2015). Interestingly, even man-made old-growth forests such as the Wamulin forest in China which was established in the fourteenth century, have been found to have a strong cultural significance. The belief that the prosperity of the forest is linked to the fortune of its people, one which is shared by many cultures, is what saved this expanse from the axe and helped with its designation as a nature reserve (Yang et al. 2021). It has thus been found that many old-growth forests and even single large old trees have been preserved around the world due to the cultural attachment the surrounding local and/or indigenous population has felt for them (Huang et al. 2020). This kind of continuing connection and even identification with old trees has certainly influenced the fact that the designation of national or even world heritage site has been bestowed on many old-growth forests (Yang et al. 2021) and should factor into the protection of further such expanses.

## Conclusion

Large old trees are organisms of great importance in the field of forest ecosystems as they are able to fix large quantities of atmosphere gases, produce oxygen and create local habitats which are unique and are not comparable to younger stands of trees. Nevertheless, climate change and the ensuing frequent forest fires, as well as logging, continue to have a global impact on the forest’s matriarchs (Fig. 4), while newer specimens that could be the large old trees of the future are becoming rare. In this work, we analyzed the literature available on old-growth forests and large old trees and reported on some ecological aspects that are fundamental not only for the health of the environment and ecosystem preservation but also with respect to human health. We also analyzed the socio-cultural benefits of large old trees, an often neglected feature that could be given more emphasis when planning novel nature protection programs worldwide. From an ecological perspective, old-growth forests are important carbon sinks both above and below the ground and continue to sequester large amounts of carbon. Large old trees also play a major role in biological nitrogen fixation contributing to its bio-availability as shown in forests of British Columbia, Canada. Moreover, they provide ideal nursing grounds for new growth as well as controlling below-ground conditions essential for tree regeneration in their immediate surroundings. Stable isotopes of both carbon (^13^C) and nitrogen (^15^N) in the soil and litter surrounding these mother trees act as markers for the spatial and temporal extent of their below-ground beneficial effects. When looking at their benefits to human health, apart from their ability to filter the air, produce medicinal volatile organic compounds, and the medicinal and culinary uses of their various parts, large old trees have a unique cellular structure which makes them ideal hosts for endogenous *fungi* which in turn are sources of specific phytochemicals with therapeutic properties. Also important to our mental and spiritual wellbeing, old trees are part of our human identity and cultural heritage, presenting us with aesthetic, symbolic, religious, and historical cues. In many cultures, particularly large trees are in fact treated with reverence. All in all, we are convinced that looking at the big picture, at all the numerous benefits provided by large old trees in terms of environmental, oceanic, ecological, therapeutic, and social-cultural impacts, could contribute to the arrest of their worldwide decline. Indeed, never has the phrase “to see the wood for the trees” been more relevant. We have sadly fragmented trees into specific economic or ecological values while forgetting that they are so much more than the sum of their parts. Perhaps the saying should now be that we have failed to see the trees for the wood.
